# Tackling physical inactivity and inequalities: implementing a whole systems approach to transform community provision for disabled people and people with long-term health conditions

**DOI:** 10.1186/s12889-024-18051-6

**Published:** 2024-02-28

**Authors:** Anna Pettican, Robert Southall-Edwards, Gina Yannitell Reinhardt, Valerie Gladwell, Paul Freeman, William Low, Robert Copeland, Louise Mansfield

**Affiliations:** 1 School of Health and Social Care, University of Essex, Wivenhoe Park, CO4 3SQ Colchester, UK; 2 School of Sport Rehabilitation and Exercise Sciences, University of Essex, Wivenhoe Park, Colchester, UK; 3 Department of Government, University of Essex, Wivenhoe Park, Colchester, UK; 4 Institute of Health and Well-being, University of Suffolk, Waterfront Building, Ipswich, UK; 5 School of Social Sciences, Heriot-Watt University, Edinburgh, Scotland, UK; 6 Advanced Well-being Research Centre, Sheffield Hallam University, Olympic Legacy Park, Sheffield, UK; 7 Division of Sport, Health and Exercise Sciences, Heinz Wolff Building, Brunel University London, Uxbridge, UK

**Keywords:** Whole system approaches, Physical in/activity, Public health, Health inequalities, Systems leadership, Care homes, Occupational therapy, Disabled people, Long-term conditions, Health and social care

## Abstract

**Background:**

Physical inactivity is a global public health priority. There are known health and well-being consequences of being inactive, and the benefits of being physically active are well established. However, there are persistent inequalities when it comes to how physically active people are, with disabled people, people living with long-term health conditions, and people residing in areas of socio-economic deprivation being particularly affected. Methods such as whole system approaches (WSAs), which are dynamic, multifaceted, and engage all relevant stakeholders, have gained momentum as an approach to address such complex public health problems. However, evidence relating to the implementation of WSAs to address physical inactivity is lacking. The aim of the Prevention and Enablement Model (PEM) was to take a whole system approach in Essex to encourage and support disabled people and/or individuals living with long-term health conditions to be more active, happier, and to live more independently.

**Methods:**

The aim of this study was to explore the enablers, challenges, and reflections associated with the process of designing and implementing the PEM. Semi-structured interviews (*n* = 12) were used to collect data from people involved in the PEM’s design, implementation and/or delivery. Data was analysed using Braun and Clarke’s reflexive thematic analysis.

**Results:**

Four themes were identified: (1) Working collaboratively: Specific enablers of time and space were identified as important in the planning and implementation of a WSA (2) Leadership and planning: Distributed and flexible leadership was identified as central to successful implementation (3) Re-orientating practice: Highlighted the transformative potential of a whole system approach and how it contrasts with conventional work practices, and (4) Reflection and learning: Informing ongoing refinements and further implementation of successful system change.

**Conclusions:**

These findings highlight the challenge and complexity of implementing a WSA that involves diverse stakeholders from across adult social care, the NHS, and the third sector. Several important enablers are identified, such as leadership and planning, and the challenges and discomfort that can arise whilst changing systems. Ongoing efforts are required to ensure that different elements of the system collaborate effectively to address inequalities in physical activity participation, through the implementation of a WSA.

**Supplementary Information:**

The online version contains supplementary material available at 10.1186/s12889-024-18051-6.

## Background

Globally, around 28% of adults do not meet the World Health Organisation (WHO) recommendations on physical activity (PA), which are to participate in at least 150 minutes of moderate-intensity, or 75 minutes of vigorous-intensity physical activity (PA) per week [1]. However, such physical inactivity is not evenly distributed, with there being particularly disproportionate levels of physical inactivity amongst populations such as disabled people, people living with long-term conditions, and people living in areas of high socio-economic deprivation [2–4], as well as it being compounded in incidences where such factors intersect [5–7]. The term *disabled people* is deliberately used in this paper to reflect the UK social model of disability and to align with the language preferences of the organisations involved in the PEM. However, critique of this conceptualisation and the complexity of language choice in this area is acknowledged [8, 9]. It is also acknowledged that there are many and varied groups who may face barriers to participation in physical activity, as there are many and varied aspects of identity that serve to construct and reconstruct marginalisation including those related to ethnicity, age, gender, socioeconomic status, sexuality, and disability [5, 7]. Moreover, it is important to note that such characteristics interconnect to reinforce inequality and marginalisation, and to ultimately influence health [10].

Trends in physical inactivity occur within the context of modern lifestyles becoming increasingly sedentary, due to motorized transport and the growing use of screens for work, education, and recreation [11]. The definition of PA adopted within this paper is “…people moving, acting and performing within culturally specific spaces and contexts, and influenced by a unique array of interests, emotions, ideas, instructions and relationships.” [12]. PA might be undertaken through sport and recreation, as well as through activities at work and at home, such as hanging up the washing, walking or wheeling to the local shops, or gardening [12, 13]. Regularly participating in PA provides significant benefits for physical and mental health [14–16]. Meanwhile, physical inactivity is one of the leading risk factors for noncommunicable diseases mortality, with people who are inactive having a 20–30% increased risk of death compared to people who are sufficiently active [1]. The associated morbidity of health disorders related to inactivity, including health-related quality of life as well as direct and indirect economic costs, represents a considerable burden on society, including health and social care systems. For example, if current levels of physical inactivity do not change, it has been estimated that 499.2 million new cases of preventable non-communicable diseases would occur globally by 2030, with direct healthcare costs of INT$520 billion [17].

There is an increasing awareness that physical inactivity is the emergent result of many interacting elements within a complex and dynamic social system [18, 19]. Indeed, the World Health Organisation’s Global Action Plan on PA suggests that whole system approaches (WSAs) are needed to address such complex interactions among the correlates of physical inactivity, which have been identified as spanning themes such as transport and environmental factors (e.g. walking infrastructure and cost/convenience of driving), societal factors (e.g. social and cultural norms, and cycling culture), and individual factors (e.g. knowledge and habit) [20]. This shift towards WSAs represents a significant move away from traditional linear models of cause and effect [21] and interventions that have historically relied on decision-making and individual agency as the primary mechanisms of change [22]. There is now broad consensus around WSAs being required to help address an array of complex public health issues [16, 21, 23–28].

## Whole system approaches to physical inactivity

A WSA to a public health challenge requires the active involvement and interaction of multiple stakeholders, including the users of services, in its design and implementation [25]. Each stakeholder or organisation is an actor in the system that both affects and is affected by it, ensuring that the system focus is suitably aligned with the needs of service users. A WSA is deemed critical to meaningfully tackling physical inactivity within the context of people’s everyday lives [18] and there is a growing interest in such approaches related to addressing physical inactivity in particular [13, 21, 26, 29]. Such collaborative working aims to achieve long-term and sustainable systems change, through bringing together stakeholders from across different organisations and sectors and working in partnership around a common purpose [30].

However, evidence of how to operationalise and implement WSAs to address public health problems is still in its infancy. The term “*whole system change”* has become ubiquitous to the point of losing meaning, often being used to describe a range of approaches and types of stakeholder involvement [18]. Bagnall et al. [26] and Bellew et al. [31] have both called for more consistency in definitions and language relating to WSA.

In Australia, the importance of co-production and purposeful engagement with system stakeholders has been emphasised as central to the successful implementation of a WSA that tackled physical inactivity [31]. A similar initiative addressing physical inactivity in the UK has advanced a Common Purpose model to explore the complexities of physical inactivity. This was co-developed by a practice team and an academic process evaluation team, which sought to support learning and implementation as part of the ongoing realist process evaluation, in acknowledgement of the complexity of WSAs [32]. This aligns with similar calls from two other whole system PA initiatives in the UK, with their implementation reflections specifically acknowledging that the involvement of service users in the design was not as prevalent as it should be [33]. The gaps in knowledge that arise from such limits in the process can make it difficult to decide how to design, direct, plan, implement, and evaluate WSAs that address physical inactivity [26, 34].

Research to date has been largely focused on exploring the specific contribution of *health* system leaders and health professionals within the context of whole systems approaches that tackle physical inactivity [18, 35, 36], rather than on the learnings acquired from designing and implementing WSAs that specifically bring together multi-sectoral actors from across a diverse and too often fragmented health *and* social care system. Doing so would encompass a much broader workforce, including local authorities and third sector organisations, alongside the National Health Service (NHS), and the involvement of service users [37]. Indeed, there is a growing acknowledgment that allied health professionals and social workers have a key role to play in tackling physical inactivity and addressing health inequalities, as part of a workforce that spans the health and social care sectors [38–41]. Furthermore, designing WSAs that bring together and support such a workforce in tackling issues such as physical inactivity aligns with agendas to develop more integrated and preventative practices across health and social care [37, 42–44]. Hurley and colleagues have already explored the value of two allied health professionals, occupational therapists and physiotherapists, in increasing PA in care home residents through the implementation of a WSA [45]. However, this implementation was limited to a care home context and there is a need for evidence on the implementation of whole systems approaches that involve a diversity of context and stakeholders, from across health and social care.

### The prevention and enablement model (PEM)

Essex, a county in the East of England, is one of 12 locations selected by Sport England’s Local Delivery Pilot (LDP) programme. The LDP is commissioned by Sport England, as a pioneering programme underpinned by a WSA, which recognises that changes are required across policy, infrastructure, culture, and communities to tackle physical inactivity effectively [33, 46]. From each of the 12 LDPs in England, there was and still is potential to generate insight and learning that can contribute to other localities and countries developing their own WSAs to tackling physical inactivity. The Essex LDP focuses on areas of high deprivation and physical inactivity in Basildon, Colchester and Tendring, where barriers to PA are extensive [47, 48]. In this study, we offer an in-depth understanding of the enablers, challenges, and reflections associated with the process of designing and implementing one particular innovative whole system programme within the Essex LDP, which sought to enable disabled people and those living with long term health conditions to move more.

The Prevention and Enablement Model (PEM) was an Essex LDP funded WSA to tackle physical inactivity and inequalities in the county. The overarching aim of the PEM was to take a whole system approach in Essex to encourage and support disabled people and/or individuals living with long-term health conditions to be more active, happier, and to live more independently [49]. It used PA as a preventative and therapeutic tool to avoid deterioration of health conditions and improve quality of life. It was envisaged that service users might also rely less on community support services, which would facilitate reductions in long waiting lists and prevent community support services and other resources from being overstretched.

The PEM was co-designed by stakeholders from across the health and social care system: a social care local authority, two NHS trusts, and a local social enterprise (Sport for Confidence CIC). The PEM deliberately used a no labels approach to avoid categorising and excluding populations. Instead, it was open to anyone who was physically inactive and wished to be more active. A working party was formed in Spring 2020 to collectively shape the design and delivery of the PEM. It was expected that service users would be directly involved in the working party, but its work took place right at the beginning of the COVID pandemic, in a time of restricted practices and with limited online mitigations. Occupational therapists with day-to-day contact with PEM service users were included in the membership of the working party, and while it could not be claimed that they could speak for people who were not there, their professional expertise did mean they could give some insight into the lives of disabled people and people living with long-term health conditions. It is an acknowledged limitation of the PEM that its design and implementation relied on such professional expertise, rather than directly involving PEM service users. In future, the service users of WSAs must be actively involved in such working party practices.

This working group later evolved into a project group from August 2020, which was initially comprised of 30 individuals from across the health and social care system, and there were also individuals from commissioning and delivery teams to reflect different parts and levels of the system. They came together around the common issue of physical inactivity and the project group worked together for over two years to design, implement, and reflect on the PEM as a WSA.

The development and design of the PEM is detailed here in response for calls for detailed descriptions of WSAs to assist with planning, design, and implementation [26]. The design of the PEM involved four distinct strands of community provision: (1) An integrated community-based strength and balance pathway that involved NHS Otago sessions [50] and ongoing ‘step-on’ provision to the social enterprise facilitating sessions in local leisure centres, (2) A programme of community-based inclusive sport sessions in local leisure centres that were designed and led by occupational therapists and provided a safe place for participants to return to and move on from in terms of PA participation, (3) A practice development programme for occupational therapists, and (4) An education programme and monthly mentoring from an occupational therapist for care home staff.

Bagnall et al. [26] have proposed 10 features of a systems approach to address complex public health problems such as physical inactivity: Identify a system; capacity building; creativity and innovation; relationships; engagement; communication; embedded action and policies; robust and sustainable; facilitative leadership; and monitoring and evaluation. A number of Bagnall et al’s [26] key features were present in the WSA that PEM implemented, such as creativity and innovation, relationships, and facilitative leadership.

This is one of the first papers to detail learnings specifically from the design and implementation of a system approach that engages diverse community stakeholders from across the health and social care system in the United Kingdom (UK), to implement preventative practices that encourage and enable inactive people to move more. The aim of this study is to explore the enablers, challenges, and reflections associated with the process of designing and implementing the PEM.

## Methods

The study detailed in this paper was a distinct strand within the wider evaluation of the PEM, which adopted a mixed-methods approach. This paper focuses on data from interviews with 12 individuals who were involved in the commissioning, funding, designing and/or delivery of the PEM.

### Participants

The study used purposive sampling [51], a form of non-probability sampling, in that participants were recruited as people who had a direct connection with commissioning, funding, designing and/or delivering the PEM. Recruitment was conducted using Essex LDP communication channels (email and mentioning the opportunity at relevant meetings). Potential participants were sent a participant information sheet and consent form. Participants were required to be willing and able to engage in an online semi-structured interview and to give informed consent. Twelve participants were recruited; two were men and 10 were women, which represented a diverse range of stakeholders who were involved in the planning and implementation of the PEM. Details of the participants are provided in Table 1. All interviews were conducted in January 2021. Informed consent was obtained from all participants before interviews commenced.

**Table 1:** Participants

Pseudonym	Employer	Role with the PEM
Eden	Local authority	Quality provision within care homes
Ashlee	Local authority	Occupational therapist, designing and delivering
Jennie	Local authority	Occupational therapist, designing and delivering
Kate	Local authority	Occupational therapist, designing and delivering
Laura	Social enterprise	Occupational therapist, designing and delivering
Debbie	Local authority	Public health registrar, designing
Christine	NHS	Commissioning and designing
Kofi	Local authority	Designing and delivering
Khalida	Local authority	Designing and delivering
James	Local authority	Designing and delivering
Nicola	Local authority	Commissioning, funding, and designing
Anca	Social enterprise	Occupational therapist, designing and delivering

### Procedures

The interview schedule (see Additional file 1) was drafted by AP, with PF, VG and GR then reviewing it and providing comments and amendments that were incorporated. The first interview acted as the pilot, with the data being deemed sufficient for inclusion and no changes being made to the schedule. In advance of their interview, participants were provided with a participant information sheet and copy of the interview schedule. It was specified that the questions in the schedule were just a guide and no specific preparation was required. To provide consistency in questioning, two members of the LDP evaluation team (GR and VG) conducted the semi-structured interviews, which are a recognised way of structuring and collecting qualitative data that requires detail and contextual information [52]. The interview questions were used as a guide rather than a rigid line of questioning, and therefore the order and phrasing were at times altered to suit the specific interview context.

Due to COVID restrictions, all interviews were conducted and recorded via Zoom, except for one interview that took place via telephone due to interviewee circumstance. For this interview, the interviewer (GR) took comprehensive notes, which were included in the analysis alongside the Zoom interview transcripts. The interviews ranged in length from 23 to 70 minutes. All the recorded interviews were transcribed verbatim by a professional transcriber. Transcripts were anonymised and then checked for completeness by AP and RSE.

### Data analysis

Braun and Clarke’s [53, 54] six stages of reflexive thematic analysis were used as a guide to ensure a rigorous and robust analysis process. The process involved two researchers (One post-doctoral - AP, and one final year PhD candidate - RSE) facilitated by qualitative analysis software (Nvivo v1.3, QSR International, Australia). One researcher (AP) acted as a primary analyst, was an occupational therapist, and had a direct involvement in the evaluation of the PEM.The other researcher (RSE) did not have an involvement and functioned as a critical appraiser, who provided perspective from not being immersed in the field of work. From working in this way together, the two researchers found that they achieved a richer and more nuanced reading of the data, rather than just seeking consensus on meaning [54]. Whilst the description here of the data analysis process implies a linearity and neatness, this does not accurately reflect the iterative cycles that the collaborative data analysis process involved. The two researchers independently read through all the interview transcripts to familiarise themselves with the data. They then also independently completed an initial coding of the data, before then coming together to discuss their coding and construct preliminary themes. The primary analyst then reviewed the coded extracts to check the themes “fitted” against each other and an original data set and a thematic analysis map was generated. Therefore, the data were analysed thematically using an inductive process driven by the study’s exploratory nature; themes were identified from the analysis rather than proceeding it [53, 54]. The coding approach was therefore collaborative and reflexive. The two researchers discussed and agreed the final themes against the coded extracts, refining theme titles and definitions, to ensure they were clear and reflective of the coded data. Together they then selected compelling extracts to illustrate each of the themes, ensuring they aligned with the research aim and captured the nuances of the data. The two researchers’ final themes were discussed and checked with two more experienced qualitative researchers (LM and GR). This led to some reorganisation of the themes, but no significant alterations to their content. This collaborative and reflexive approach to analysis was felt to be central to maintaining rigour within the analytic process and is something the researchers would employ again.

## Findings

Through the above methodology and data analysis process, four themes were identified that illuminate distinct areas of learning relating to the design and implementation of the PEM: I) working collaboratively, II) leadership and planning, III) re-orientating practice, and IV) reflection and learning. These four themes are presented below in sections, although it is acknowledged that they overlap. Each of the themes is illustrated with verbatim quotes.

### Working collaboratively

Across the interview participants there was a recognition that working well together was an important enabler to planning and implementing whole system change. However, this was not as simple as just coming together to work collectively on an initiative, there was also an emphasis on those involved needing to be open to, and tolerant of, different perspectives that may differ quite significantly from their own. Such collaborative working was enabled by the provision of a progressive and ‘safe space’ for challenge and reflection. Participants referred to both the nature and value of working together:

*“I think what’s been key really is collaboration… It’s having those regular meetings, the open forums to talk through ideas with other professionals. I think has really been key to being successful because it allows it to be more… I often think when you get an argument, you have to test your argument with a group of people because otherwise… You shouldn’t feel threatened by pushback because it’s the only way that you’re gonna make your argument bulletproof. It’s to have it being basically stripped down by the people in the room to try and investigate what you’re trying to actually achieve. And that’s how you’re able to come up with a good idea.” - James*.

For some, working collaboratively was a new and different approach– a welcome shift away from more conventional and siloed ways of working, as they re-focused on, and came together around, the common purpose of working with people to address physical inactivity:

*“…it is looking at it in a different way and I think um. some of my work that has been within my team outside of PEM is actually looking at us as a community of work force, that we’re not just silo organisations here, we’re working for that person who is everyone’s outcome, everyone’s aim, goal, you know, the residents essentially are all of our interest and whether or not we have lots of organisations round that table its. its irrespective of that. We’re all working jointly, collaboratively, so I think it is working differently” - Ashlee*.

Another enabler to such collaborative working that interview participants emphasised, was the need for sufficient time and space to allow new partnerships and relationships to develop, allowing everyone to understand their role in the collective effort. This was again a point where collaborative systems working was contrasted against conventional ways of working that were perceived as less collaborative:

*“I suppose at its heart, PEM is a partnership. And again, we talk about collaboration partnership working quite a lot in Social Care, but it’s like anything. It’s on a scale where some of the collaboration is, you might have a couple of meetings with partners and share views and come up with something. But this is definitely a collective effort. I think if one of the numerous partners that were involved weren’t, it wouldn’t work. So actually I think this is a really, again, the fact that it is different and it is a genuine partnership. And it has taken a while to build those relationships at the start and for partners to really kind of understand and see their role in the success of this and feel like they are part of it and we’re all equal partners at the table. So yeah, I think the fact that it is a genuine collective effort, and the collective, not just effort in the shared responsibility of delivering on this, but everyone is completely signed up to the vision of what we’re trying to achieve here and is doing what they can to make sure that we’re getting the most out of it.” - Khalida*.

Having system leaders directly and actively involved was also identified as an enabler of collaborative working, as part of successfully engaging and communicating with diverse stakeholders from across the system. Although it was also recognised that challenges and some discomfort might be involved to progress such system working:

*“But in terms of the actual delivery of PEM in terms of the project part, I think it has been really good having those system leaders involved in the conversations. Frustrating, but good. Annoying, a lot of the time, but worthwhile because I guess I’ve learned that they have to be around the table. If we’re gonna influence, then we need to make sure that at every level people are hearing what’s happening. So I think it’s important that there’s buy-in from those senior leaders in Adult Social Care as to what we’re doing in order to make it work.” - Anca*.

Additionally, although the involvement of service users in the design of PEM was acknowledged as a challenge, due to COVID restrictions, the importance of collaborating with the service users of system approaches was emphasised as important in pursuit of working collaboratively:

*“So we are working with… I guess, all the way from the people that deliver day-to-day on the ground to the people that commission the services in the first place. But most importantly for me, it’s about working with the people that receive a service. So in all of the workstreams (and this might not be everybody’s approach, but definitely any [social enterprise] member of staff), it kind of starts with the person, and we build from there.” - Anca*.

### Leadership & planning

The learning illuminated in the *working collaboratively* theme was further extended in the *leadership and planning* theme. This emphasised the need for leaders to identify and involve diverse stakeholders from across the system as an important enabler of system working. Furthermore, the need for a distributed leadership style was emphasised as being central to successful implementation. This strategy helps to avoid tensions with sustainability when particular people are central to a successful initiative and the risk that momentum, or continuity, could be disrupted if they depart:

*“I think key has been incredibly strong leadership and advocacy from [name redacted], and also I think, incredible energy and enthusiasm from [name redacted]. I think, I really think those two carry the whole thing, which I think is a hugely important… But obviously for system change, we need to turn that into bringing everybody else along as well. So, you know, you can’t just rely on one or two great people, because then if you lose them or something happens the whole thing can peter out. So I think, like I said, that’s where [name redacted] I think is a fantastic advocate, and she’s worked incredibly hard to raise the profile of PEM and the opportunities.” - Debbie*.

Specific leadership qualities and skills were also emphasised. For example, the need to not just provide leadership in terms of a vision and change, but also to successfully mobilise the whole PEM team towards a common purpose, in order to bring *“everyone else along as well”.* Furthermore, leadership qualities of advocacy and raising the profile of PEM as a progressive initiative were highlighted, as part of achieving change across the broader system.

Further expanding on distributed leadership, was an emphasis on the importance of co-ordination and planning. It was important to provide clarity around everyone’s roles and responsibilities, helping to engage them in working together towards implementing systems change. This is another point at which these first two themes overlap:

*“I suppose, there’s probably multiple different elements. But I suppose, at the start, from a project delivery point of view, we were, we collaboratively, as the different workstreams came up with what it was that we wanted to do as part of that work, what success looked like to us, what we wanted to achieve. Each workstream did the like logic model and took it through a very logical process as to why are we doing this? What is this actually going to look like? And I think that helped people see, you know, feel like they were part of it, and just be really clear on what the roles and responsibilities were.” - Khalida*.

However, alongside the need for leadership qualities that provide distributed working and clarity, there was an emphasis on allowing flexibility. Such flexibility enabled a shift away from conventional ways of working, towards a more progressive, organic, and wider systems change approach. Within the quote below, the implications this had for transforming existing commissioning processes is particularly emphasised as an example:

*“Well I think everyone’s had a shared vision, so it has naturally developed well. I think that what’s enabled that development is that whilst the brief for PEM is clear, it allows for enough flex for its natural growth. So I think it’s been able to develop quite naturally. I think the partners be it, [name redacted], [name redacted], the clinical commissioning groups have all worked well, extremely well together because they have been under that shared objective and I think as well it is an entirely new way of commissioning, I think at Essex, because typically the brief would be given to the provider, the provider would have said yes we can do it from that, or no we can’t... whereas now, actually it’s a commissioning approach which is, I guess, it is new and we are working together with the provider which isn’t typically done” - Kofi*.

### Re-orientating practice

A key enabler of the implementation of the PEM was that it led to a re-orientation of professional practice within the wider system, relating to tackling physical inactivity and working within a more preventative, public health frame. Indeed, many participants noted that PEM had gone beyond previous approaches to address physical inactivity through also focusing on education and support. This was perceived to contribute to a shift in culture, rather than just the provision of equipment or ad-hoc exercise classes. This is a significant transformation for the UK health and social care context, which has conventionally worked in a way that is reactive rather than preventative:

*“Yeh, so I don’t think there has been that real commitment before, to looking at prevention in a practical approach really. I’ve heard of lots of similar, potentially, activities which have gone on in care homes where they have supplied hoola-hoops and boccia kits maybe but actually what we’re doing now is actually trying to change culture, which I think is the huge different piece that PEM is on in terms of this journey. We are trying to change the culture of people who are not even in receipt of adult social care yet, which I think is the difference as well. So, yeh I think. for me really it is seeing that growing commitment to prevention and word is spreading as to what we are trying to do and everybody wants to know and understand as to how they can get involved in it” - Kofi*.

In addition to enabling change in overall culture, there was also evidence of prevention within individual practices. For example, Adult Social Care Occupational Therapists developed their own sessions to think and talk together about how physical activity might be used therapeutically within their practice. In this sense, the PEM stimulated conversations and actions, rather than just being start and end points:

*“So the idea of the PEM model is that we are going to help prevent people from their medical conditions worsening. So by engaging in physical activity, it’s gonna improve people’s well-being mentally and physically and, therefore, it will reduce the demand on the NHS. So currently, I’ve got a client that needs… If we didn’t intervene, he would probably end up in a mental health unit because he’s developing symptoms of psychosis, which we have identified through him doing activities. So we’ve identified this now. So we’re able to get the appropriate helpin now to prevent admissions.” - Laura*.

Furthermore, there were noticeable positive shifts in people’s capabilities and confidence to utilise PA and address physical inactivity within their practice. This was not just reserved to occupational therapists, but also across the practice of other health and care professionals involved in the PEM:

*“Creating practice-based learning opportunities to ensure that physical activity is at the heart of re-thinking social care practice and in that I guess is how we transform the ways of working and improve confidence and capability across the workforce. So whether that is social workers, that is occupational therapists, whether that is care home staff, whoever it may be, at whatever point, whether that is carers… it is anyone’s, but it is really about improving that confidence and capability to use physical activity as a tool–* Kofi.

### Reflection & learning

Ongoing reflection and learning during the implementation of the PEM informed refinements and further implementation of successful system change. Critical to such learning was appreciating that undertaking system change on this scale would present challenges and barriers throughout the process, requiring perseverance, and resilience. In the quote below the challenge of trying to work across different health and social care sectors towards prevention and the need for tenacity is discussed:

*“Yeah, so GP’s […] are really difficult to get them in. You try and give them a flyer, and you know the flyer’s just gonna sit in the reception area. They don’t really take it on board and discuss that with other people. So that would have to be a whole workstream, I think, on its own. I’ve also tried to put the fliers out in NHS venues. And there, I’ve been told that I can’t put them in there because they aren’t an NHS group, which makes it difficult because then everybody becomes reliant on the NHS and you don’t break that circuit. But I then spoke to the communications department of the NHS… and they said that they were gonna change that and then they then put the flyer in there. But it’s things like that where, if I didn’t pursue it, it would have stopped and it wouldn’t expand.” - Anca*.

The scale and diversity of the evaluation approach that had been undertaken was also highlighted as an important aspect of enabling learning and workforce development, particularly when looking to evidence the impact of implementing a whole systems approach:

*“In my experience with Social Care, there’s a very varied approach to evaluating either big, year-long pilots such as this, or even short-term contracts that we commission providers to oversee. It is quite varied according to their commissioners that are involved. So we have different types of monitoring and data reporting to see how things work, but actually, I personally haven’t worked on anything with an evaluation of this scale and it’s varied nature. So it’s looking at obviously, a lot of it is around the qualitative aspect, what data or what the quantity of the data that we’re capturing and what that shows in terms of maybe cost benefits, but really bringing in the voice of the resident and particularly our work force. So a big part of this is how we’re hopefully changing some of their mindsets and increasing the competence of our workforce to work in this way. And the way that we’re capturing, you know, doing interviews such as this and learning throughout, and the learning logs that we do, case studies that we’re coming up with just to kind of really show I think that overall picture of the impact of this. So it’s not just from a financial point of view. It’s not just from the impact that we’re seeing on maybe practice changing. It’s a real, I suppose, holistic evaluation. So I’d say that is very different in a good way.” - Khalida*.

However, some challenges associated with evaluating and learning from system change were noted. There was a lack of understanding and agreement around how to best evaluate a WSA. For example, some stakeholders were drawn to methods they had previously experienced and used in smaller scale projects. Furthermore, there was a challenge to ensure the evaluation met the needs of a diverse range of stakeholders:

*“So I think the evaluation approach hasn’t been clear and also, because it’s sat within this already rigid restraint, or sort of rigid constraints of LDP. I don’t know if it necessarily, from my perspective anyway, fits PEM particularly well. And I think that the outcome of that is we’re still, we’ve got our short-term metrics of what we think success looks like, but we’re trying to figure out, how do we collect those without making, being a double burden. Because obviously we have to collect stuff for PEM evaluation through the [name redacted], but we also need stuff for the actual project and the service delivery to see if it’s working.” - Debbie*.

## Discussion

This study identified key enablers, challenges, and reflections associated with the planning and implementation of the PEM; an innovative multi-faceted WSA to enable people who are inactive to move more through engaging multi-sectoral actors from across the health and social care system. As the practice of designing and implementing WSAs to address physical inactivity proliferates, it is important to explore what is being learnt by whom, where, and in what circumstances, and for such knowledge to be shared [55]. Indeed, the need for evidence to inform the operationalisation of WSAs has been acknowledged within the literature [26, 31, 34]. To address calls for descriptions of WSAs [26], this paper has also provided a detailed description of the PEM to further inform the future design and implementation of WSAs that address physical inactivity. To date, literature seeking to better understand the design and implementation of WSAs has been largely focused on the NHS and statutory health system leaders [17, 26, 27]. Significantly, this is one of the first papers to specifically outline learnings from a WSA that engages community stakeholders from across the UK health *and* social care system, encompassing both the statutory and third sectors.

The *leadership and planning* theme highlights the centrality of clear leadership and planning as an enabler within the design and implementation of a successful WSA. The existing knowledge base relating to leadership and WSAs emphasises a distinct leadership style, which does not simply provide a single driving force for action, but rather a distributed leadership approach. Such an approach advocates for and enables the involvement of diverse, wider system stakeholders and provides a catalyst for change through collaboration [35]. Additionally, it requires long-term commitment and ongoing leadership to ensure sustainability [13]. The reflections provided in this study as part of the *leadership and planning* theme support these assertions, with specific reference to a collaborative and facilitative style, as well as aligning with literature that has emphasised adaptability and flexibility in leadership [34].

The design and implementation of the PEM concurs with assertions that WSAs necessitate collaboration between multi-sectoral stakeholders, including health, social care, not-for-profit and voluntary organisations, to maximise impact [29]. Such diversity in thinking, planning, and action aligns with calls for inter-disciplinarity in order to garner understandings of health promotion that extend beyond epidemiological knowledge to other approaches used by health and social care professionals when seeking to address physical inactivity [41].

Notions of diversity should therefore be noted as including various forms– in terms of the different sectors that should be involved in WSAs, as well as the individuals and their various roles (commissioning, planning, implementing, and service user) that were highlighted in the *working collaboratively* theme. Furthermore, diversity in thinking is apparent in the study’s *re-orientating practice* theme, which provides valuable new knowledge by highlighting the role of WSAs in changing the way system stakeholders think, and then re-orientating their work and practices accordingly. This theme therefore provides evidence for the assertion that WSAs provide a ‘mindset’, or cognitive framework, which enables stakeholders to make sense of complex systems, become better problem solvers, and take action accordingly [31]. This supports literature that suggests diversity in system stakeholders, and consequently thinking, are integral to successful and impactful WSAs [29] and should not be overlooked within implementation processes [23–25].

The *re-orientating practice* theme finding is also particularly significant when considering the enormity of plans to re-orientate a health and social care workforce to work within a more preventative frame [56]. It should therefore inform continued efforts to develop a health and social care workforce that has the capability, skills, and confidence to promote PA [36, 41]. This is a point at which the *re-orientating practice* and *working collaboratively* themes overlap, in terms of the practices involved with ensuring diverse system stakeholders are provided with enablers such as sufficient time and (safe) space to explore and ‘test out’ new ideas and ways of working together.

Conversely, the *working collaboratively* theme also illuminates the challenges and discomfort that can occur when bringing together diverse and diffuse actors from across the system. Whilst a shift from siloed towards integrated working has been emphasised as a necessary component of WSAs [25], the challenges and discomfort that can occur during implementation are absent within the literature on WSAs to date. Perhaps because it has predominantly been theoretically rather than practically orientated [25, 32]. A shift from siloed to integrated working is emphasised as a necessary component of WSAs [25]. Therefore, the PEM disrupted conventional ways of working and progressed a novel approach, involving both the statutory and third sectors, to design and implement preventative practices that encouraged and enabled inactive people to move more. *Leadership and planning* is suggested as a protective factor to mitigate against the challenge and discomfort that some participants expressed in the working collaboratively theme.

Although service users were not part of the PEM working party, the importance of finding ways to engage with and involve service users in the everyday delivery of the PEM was also emphasised within the *working collaboratively* theme. This aligns with literature that has detailed the use of co-production and involvement of service users as features of successfully implementing WSAs that address physical inactivity [31, 33]. This is particularly important when service users are from marginalised and under-represented populations, such as the disabled people and people with long-term conditions, as included in the PEM [55].

Within the *reflection and learning* theme there was again overlap with the *working collaboratively* theme, in terms of the challenges and barriers that can be encountered within system working, and the collective learning and refinements that are necessary. The role of evaluation was specifically emphasised within this theme, although the risk of the evaluation approach being too rigid was discussed and confirmed in alignment with previous research [34]. It is important for evaluation to be clear, tailored, and flexible to meet the needs created by working in a WSA. Monitoring and evaluation has been identified as a key feature within system approaches, as mechanisms that provide ongoing feedback, to inform refinements and ultimately system change [26].

### Limitations

The sample was self-selecting and represents the experiences related to one specific WSA based in a county in the East of England. The circumstance of one participant meant that their interview was noted rather than recorded, which may impact how they were represented in the findings.

### Recommendations for practice, policy, and research

This novel research adds to a growing body of evidence that is concerned with how to operationalise WSAs to address complex public health problems. From the study’s findings the following recommendations are made:

1. Participatory approaches should be used with service users to ensure that the design, implementation, delivery, and evaluation of WSAs remains relevant to their lives and needs and is ultimately successful in enabling their physical activity participation.
2. A distributed leadership style should be operationalised during the planning, implementation, and delivery stages of WSAs, and a common vision and purpose developed.
3. The roles and responsibilities of stakeholders should be clearly defined during the design, implementation, and delivery of WSAs, but with flexibility for them to evolve.
4. In support of broader service user health and wellbeing outcomes, WSAs addressing physical inactivity should ensure that they have a focus on prevention, in addition to promoting physical activity participation.
5. Future work should include clear and detailed descriptions of WSAs, so that others seeking to design, implement and deliver them can use such information to inform their own WSA design and implementation processes.

If adopted, the above recommendations could benefit a number of stakeholders in different ways and via various mechanisms. For example, recommendations one and four could ensure service users, and particularly disabled people and people living with long-term health conditions, feel a sense of ownership in the approach and their own care, which might enhance feelings of agency and motivation, and thereby contribute to their PA participation and wellbeing. Similarly,the second and third recommendations ensure a broad range of system leaders and deliverers are involved in WSAs, who then in turn have clearly defined roles and responsibilities and clarity of other stakeholders whom they connect with for insight, resources, and support. The final recommendation would help system leaders and researchers to replicate good practice and develop effective ways to design, implement, and evaluate WSAs with a preventative focus to enhance PA participation and wider health outcomes.

## Conclusions

This is one of the first studies that has identified enablers, challenges, and keys areas of learning that shape and inform the planning and implementation of a WSA to address physical inactivity, which involves stakeholders from across the health *and* social care systems. The study’s findings support research that has identified the importance of working together with diverse system stakeholders [23–25] and the distinct leadership approach that is required for the successful planning and implementation a WSA. The study has provided important new knowledge about the challenges and discomfort that can occur during the implementation of WSAs that address physical inactivity. In addition, the study has added to and extended the conclusions of other studies which contend that service users and the wider health and social care workforce can, and should be, actively involved in the planning and implementation of WSAs that address physical inactivity [27, 29–31].

### Electronic supplementary material

Below is the link to the electronic supplementary material.

Supplementary Material 1

## Data Availability

The data that support the findings of this study are available on request from the corresponding author, (AP). The data are not publicly available due to their containing information that could compromise the anonymity of research participants.

## Abbreviations

LDP: Local Delivery Pilot
PEM: The Prevention and Enablement Model
PA: Physical Activity
WSA: Whole System Approach
