# Supplementary material for: Tackling physical inactivity and inequalities: implementing a whole systems approach to transform community provision for disabled people and people with long-term health conditions
Source: BMC Public Health. 2024 Feb 28;24:636. doi: 10.1186/s12889-024-18051-6 (PMC10903012; doi:10.1186/s12889-024-18051-6)
Supplement: Supplementary file 1 — Supplementary Material 1 [file 12889_2024_18051_MOESM1_ESM.docx]

**Copy of interview schedule**

Evaluating physical activity behaviour change in Essex: Part of the local delivery pilot

QUALITATIVE DATA COLLECTION (PEM – EVALUATION FRAMEWORK): SAMPLE QUESTIONS

Thank you for taking time to speak to us today. We would like to ask you some questions about PEM. Have you had a time to read the information sheet and consent form? Do you have any questions before we start?

1. Please could you start by telling me about your background and role in relation to the PEM project? For example: length of involvement, type of involvement, reason for being involved, how you became involved, which workstreams are you involved in
2. Could you please describe the aims and objectives for PEM overall and/or any specific workstreams you are involved in?
3. Could you please tell me how PEM and/or its workstreams meet the strategic goals/ business plan of Adult social care, and the wider system (consider wider determinants of health (education, work environment, unemployment, health care services, housing)? Why is it needed? And why now? For example: Early Intervention, Prevention, Poor Health, Covid19 lens
4. Could you please describe any differences in approach of PEM and/or its workstreams compared to existing ways of working that you know of in adult social care and other health and care settings (consider wider determinants of health (education, work environment, unemployment, health care services, housing)? For example: systems thinking, hardwiring physical activity into the system, collaboration, engaging unusual individuals, workforce and culture change
5. Could you tell me about the key stakeholders/partners in PEM overall and/or any workstreams in which you are involved? For example: leaders, commissioners, deliverers, organisations, people, families, informal carers and workforce; their roles;
6. Could you describe the key success factors and what has worked well in the:
   1. Development of PEM overall and/or specific workstreams? For example: Local Delivery Pilot on Page priorities and ways of working (e.g., understanding the needs, strengths and asset based, personalised/bespoke/unique solutions, opportunities and resources within systems; collaboration; data & insight)
   2. Delivery of PEM overall and/or specific workstreams? For example: implementation, adoption, fit to different context
   3. Impact of PEM overall and specific workstreams? For example: outcomes, QoL, happiness, reach, effects at system level (e.g., prioritisation of physical activity, realignment of budgets, changes to policy/practice) and individual/community (e.g., increased physical activity, improved mood, health and wellbeing); mechanisms/pathways to impact (why/how)
7. Could you describe the key challenges and how these have been or could be overcome in the:
   1. Development of PEM overall and/or specific workstreams? For example: LDP on Page priorities and ways of working (e.g., understanding the needs, opportunities and resources within systems; collaboration; data & insight)
   2. Delivery of PEM overall and specific workstreams. For example: implementation, adoption, fit to different context
   3. Impact of PEM overall and/or specific workstreams. For example: reach, effects at system level (e.g., prioritisation of physical activity, realignment of budgets, changes to policy/practice) and individual/community (e.g., increased physical activity, improved health and wellbeing); mechanisms/pathways to impact (why/how)
8. Could you please describe the potential of PEM overall and/or specific workstreams over the next two years and factors that will influence its development? For example: impact on system, community or individual, scale up, reach, replicate (geographical spread), Covid19, technology
9. Could you please describe how the approach/ principles of the Local Delivery Pilot have been/could be potentially adopted across your organisation and/or in the workforce? For example: ABCD, working with local passionate people, distributed leader-ship and community empowerment, co-design and co-produce, sharing and learning, embracing innovation and risk, network and collaboration and distribution
10. Is there anything else you would like to say about PEM overall or its specific workstreams and its whole systems approach to tackle physical inactivity?

Many thanks for participating.
